# Misdiagnosis of vasitis: a potential diagnostic pitfall with computed tomography

**DOI:** 10.1186/s12610-022-00168-6

**Published:** 2022-10-11

**Authors:** Yi Hong Li, Zhon min Huang, Ji kuen Yu, Yi Sheng Lin, Chao Yu Hsu, Min Che Tung

**Affiliations:** 1 Division of Urology, Department of Surgery, Tungs’ Taichung MetroHarbor Hospital, No.699, Section 8, Taiwan Boulevard, Wuqi District, Taichung City, 43503 Taiwan; 2 Division of general surgery, Department of Surgery, Tungs’ Taichung MetroHarbor Hospital, Taichung, 43503 Taiwan

**Keywords:** Vasitis, Incarcerated inguinal hernia, Computed tomography, Diagnostic laparoscopy

## Abstract

**Background:**

Vasitis is a rare condition that may be challenging for the clinical practitioner. Sometimes it is misdiagnosed as incarcerated inguinal hernia; thus, patients end up receiving unnecessary surgery. Compared with the traditional approach with only sonography, the more recent introduction of computed tomography in the diagnostic process has provided higher quality imaging and more detailed anatomy. Consequently, some urologists advocate the efficacy of computed tomography in the differential diagnosis of difficult cases.

**Case presentation:**

We present the case of a 23-year-old male who suffered from right inguinal pain and swelling. His scrotum ultrasound showed multiple tubular structure dilatation within the subinguinal area and no testis torsion. The initial diagnosis was a right inguinal hernia. Computed tomography supported that initial diagnosis, and we presumed the lesion represented a herniation of the omentum with mesenteric vessels. Since there was a suspicion of hernia incarceration, the patient underwent diagnostic laparoscopy, which did not reveal herniation, but only erythematous reaction and swelling over the right spermatic cord. Following a final diagnosis of vasitis, he received empirical antibiotic treatment and his symptoms entirely resolved.

**Conclusions:**

Even though computed tomography can provide thorough imaging of the urogenital system, the contrast enhancement within vessels and inflammatory organs can still be misleading in the diagnostic process.

## Résumé

**Contexte:**

La déférendite est. une maladie rare qui peut présenter des difficultés pour le praticien. Parfois, elle est. diagnostiquée à tort comme une hernie inguinale incarcérée; ce qui amène les patients à subir une intervention chirurgicale inutile. Par rapport à l’approche traditionnelle avec la seule échographie, l’introduction plus récente de la tomodensitométrie dans le processus diagnostique a fourni une imagerie de meilleure qualité et une anatomie plus détaillée. Par conséquent, certains urologues préconisent l’efficacité de la tomodensitométrie dans le diagnostic différentiel des cas difficiles.

**Présentation du cas:**

Nous rapportons le cas d’un homme de 23 ans qui souffrait d’une douleur et d’une tuméfaction inguinales droites. L’échographie du scrotum a montré une dilatation de multiples structures tubulaires dans la région subinguinale, sans torsion du testicule. Le diagnostic initial a été une hernie inguinale droite. La tomodensitométrie a confirmé ce diagnostic initial, et nous avons supposé que la lésion représentait une hernie de l’épiploon avec des vaisseaux mésentériques. Comme il y avait une suspicion d’incarcération de hernie, le patient a subi une laparoscopie diagnostique, qui n’a pas révélé de hernie, mais seulement une réaction érythémateuse et un gonflement du cordon spermatique droit. Après un diagnostic final de déférendite, le patient a reçu un traitement antibiotique empirique et ses symptômes ont été entièrement résolus.

**Conclusions:**

Même si la tomodensitométrie peut fournir une imagerie approfondie du système urogénital, l’amélioration du contraste dans les vaisseaux et les organes inflammatoires peut encore être trompeuse dans le processus diagnostique.

**Mots-clés:**

Déférendite hernie inguinale incarcérée tomodensitométrie laparoscopie diagnostique

## Background

Vasitis is an infection or inflammatory condition of the vas deferens [1]. Vasitis nodosa refers to a chronic type of infection of the vas deferens with mild to asymptomatic disease [2]. Acute vasitis is a benign disease, and most patients only require antibiotic treatment. However, it is challenging for clinicians to differentiate it from incarcerated inguinal hernia [3]. The two diseases share similar characteristics, such as the acute onset, palpable lump within or below the inguinal area, and tenderness. Previously, sonography was used for diagnosis, but its sensitivity and specificity were not clarified. Some studies have suggested that a computed tomography (CT) scan should be used for a definitive diagnosis whenever the sonography report is equivocal [4]. However, fat stranding between the spermatic cords can mimic an incarcerated bowel loop on a CT scan, making it difficult to obtain a correct diagnosis.

## Case presentation

**Fig. 1:**
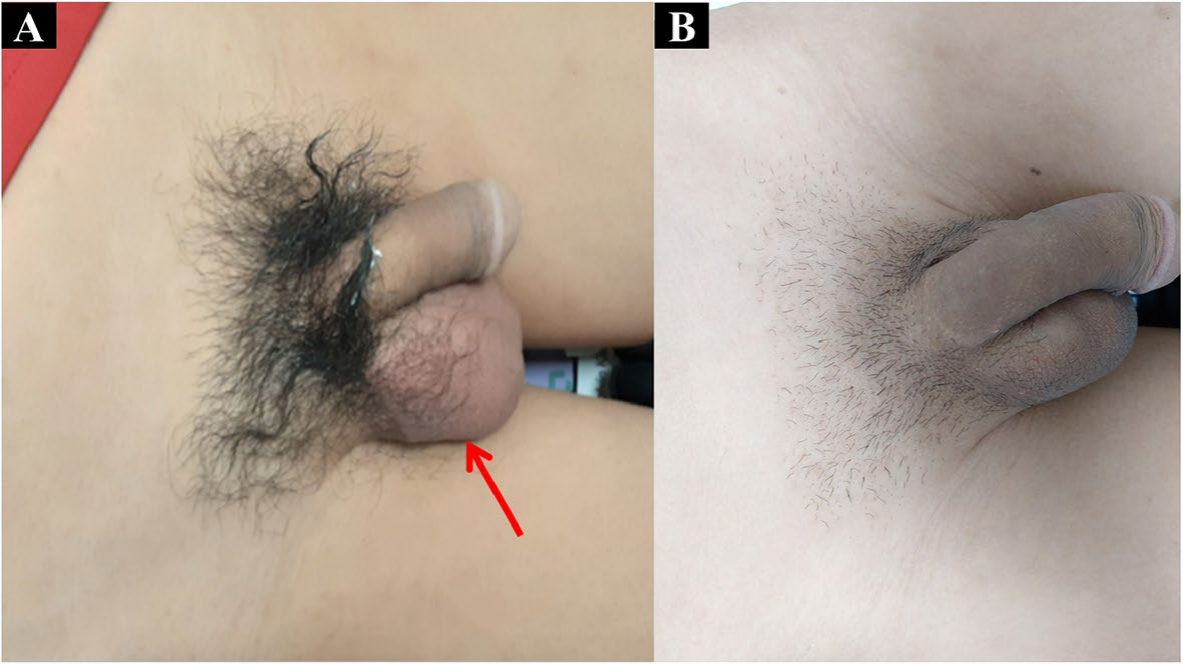
**A** Initial presentation of the right scrotum with erythematous reaction and swelling (red arrow). Suprascrotal and inguinal areas are also tender. **B** Follow-up 3 weeks later. Complete resolution of signs

**Fig. 2:**
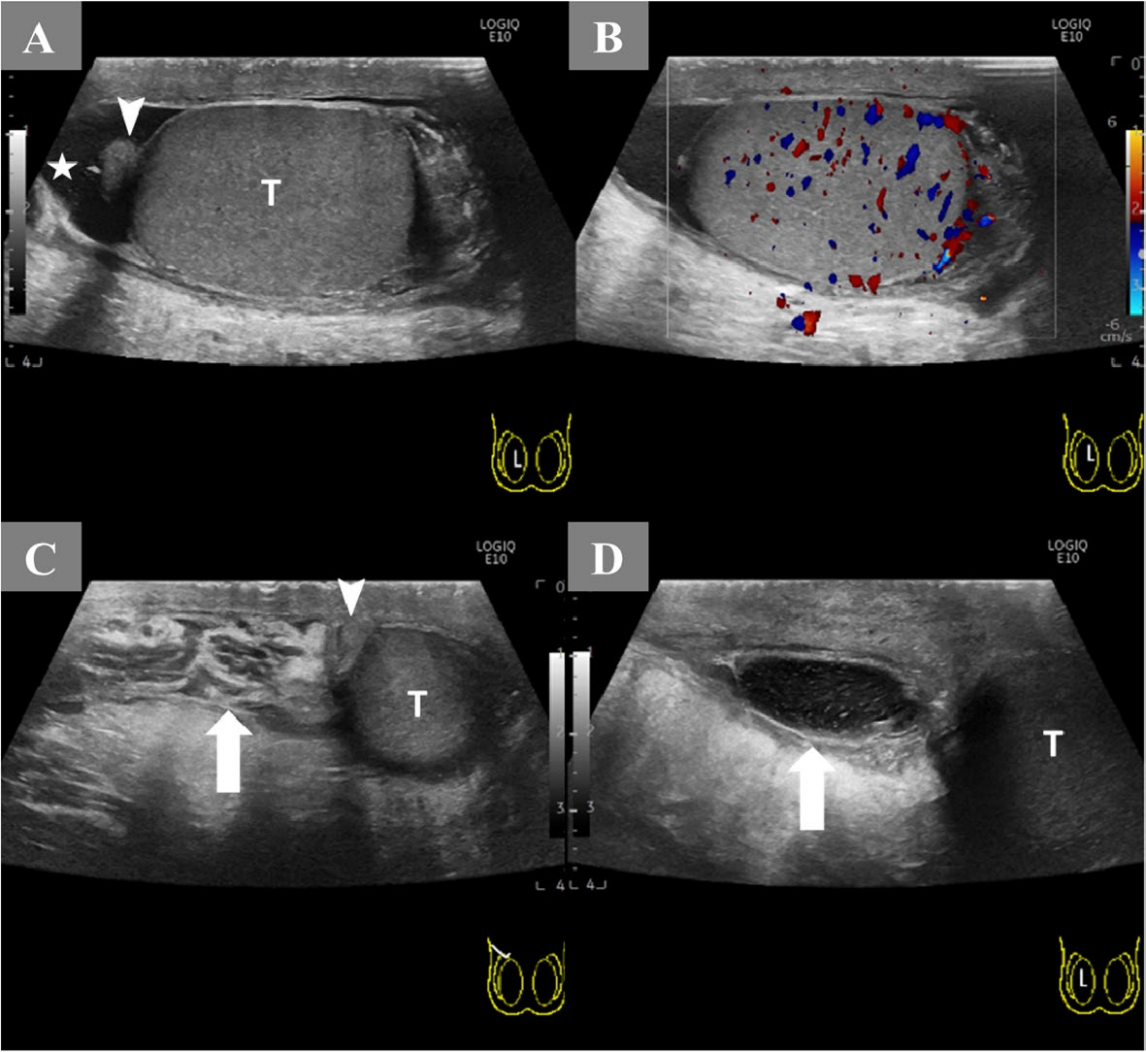
**A** Right scrotum in longitudinal view: The testis has a homogeneous texture (T) with mild hydrocele accumulation (star). The appendage above the testis is the head of the epididymis (concave triangle). **B** Normal blood flow within the right testis. **C** The transverse-oblique view above the right scrotum: multiple heterogeneous tubular-like organs with central hypoechoic but surrounding hyperechoic wall (white arrow). The maximum diameters are less than 2 mm. **D** Lateral side view above the right scrotum: well-circumscribed oval lesion with mixed-texture contents

**Fig. 3:**
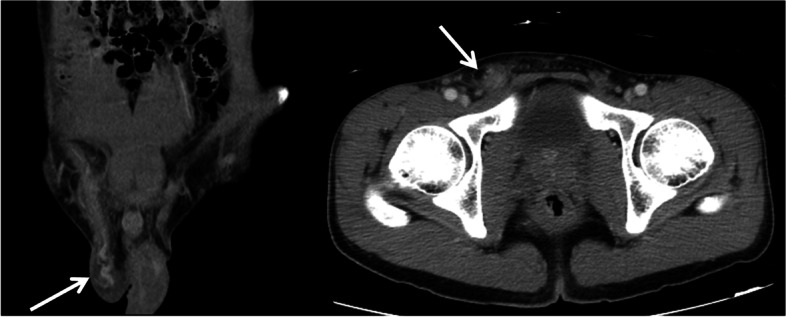
Contrast-enhanced CT. The coronal view shows multiple continuous vessels extending from the abdomen into the right inguinal area with surrounding fat stranding. Axial view shows right inguinal hernia. The white arrow highlights the equivocal lesion. Incarceration due to an ileal bowel loop with omental vessels cannot be excluded based on these images

**Fig. 4:**
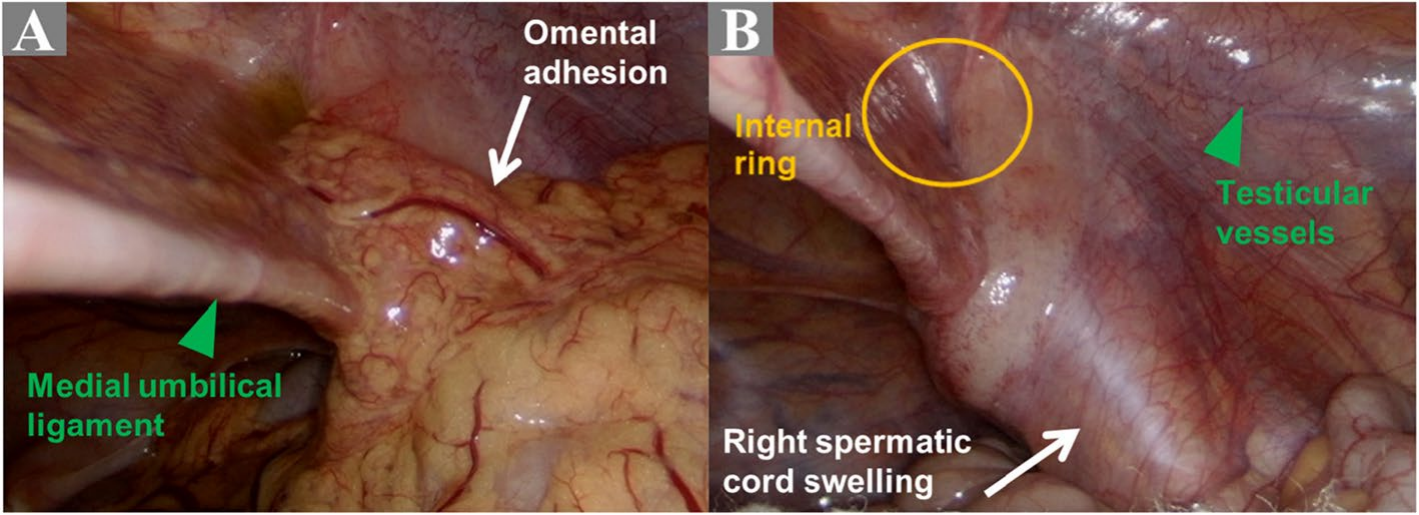
**A** Diagnostic laparoscopy reveals the ileum and omentum with congested mesenteric vessels adhered to the right internal ring. **B** After removing the omentum, there is significant right spermatic cord engorgement and an intact internal ring

A 23-year-old man presented rapidly worsening subacute pain and swelling in the right inguinal area. This had lasted for 4 days before admission. There was no history of dysuria, nausea, vomiting, or bowel habit change, and his visual analogue scale (VAS) pain score remained below 4. Moreover, the patient denied any history of systemic disease or previous surgery. His history was also negative regarding weight-bearing, chronic cough, trauma, or strenuous exercises before symptoms onset. Notably, the patient had no family history of testicular torsion. Physical examination showed right subinguinal erythema and tenderness. However, Prehn’s sign was negative, and there was no swelling of the testis and epididymis bilaterally (Fig. 1A). Inguinal hernia, infectious diseases such as orchitis or epididymitis, and an atypical presentation of testicular torsion were considered as possible differential diagnoses. A scrotal Doppler echography was performed, which revealed intact bilateral testicular blood flow and homogeneous testicular content (Fig. 2A & B). Mild hydrocele was noted. However, an indeterminate hypoechoic lesion was shown above the right epididymis, with multiple tubular structures and reactive fluid accumulation (Fig. 2C & D). The diameter was not greater than 2 mm. Valsalva manoeuvre during the ultrasound could not be performed due to the poor cooperation of the patient. A right inguinal hernia was suspected, and incarceration was not ruled out. Laboratory studies showed increased white blood cell count (1.2 × 10^3^/μL; reference range, 4–10 × 10^3^/μL) and elevated C-reactive protein levels (CRP; 3.21 mg/dL; reference normal range, < 0.3 mg/dL); however, beta-human chorionic gonadotropin (beta-HCG; reference normal range < 0.16 ng/mL) and alpha-fetoprotein (AFP; reference normal range < 8.78 ng/mL) levels were within the normal limits. Urine analysis revealed microscopic hematuria without pyuria. CT showed a segmental ileal loop within the right inguinal area and multiple hyperdense tubular organs incarcerated into the right scrotum (Fig. 3). Accordingly, omental incarceration was suspected, and diagnostic laparoscopy was performed. During the operation, we noted bilateral intact internal rings, while swelling and erythematous reaction over the right spermatic cord were noted, along with greater omentum adhesions (Fig. 4). Postoperatively, the patient had only one episode of fever, which reached 38.2 °C. Consequently, the diagnosis was made of vasitis. Initially, he was treated with ceftriaxone as empirical antibiotic treatment, then switched to cefazolin plus gentamicin on postoperative day 3. The patient was discharged on postoperative day 5, with the prescription to continue the antibiotic treatment (cephalexin) for seven more days. At subsequent follow-up visits, he denied any discomfort. No erythema, swelling, or tenderness was found during physical examination (Fig. 1B).

## Discussion

Vasitis can be classified as either an acute infection/inflammation or a chronic disease. The chronic condition, also known as vasitis nodosa, was first described in 1943 by Benjamin [1]. The histopathological examination may reveal a narrow vasal wall, thickened muscular coat, and evidence of inflammation over the surrounding tissue, such as fibrosis and lymphocyte infiltration [2]. Vasitis is a benign disease that can be either asymptomatic or present as a protruding mass with focal tenderness over the inguinal area. It is usually self-limiting and resolves spontaneously.

The pathophysiology is associated with obstruction or injury to the vas deferens, which is a complication of vasectomies. Indeed, post-vasectomy pain syndrome is associated with chronic vasitis [5]. In a retrospective study of 11 patients, Clavijo et al. [6] demonstrated other risk factors for vasitis, such as prior herniorrhaphy, prostatectomy, and possible HIV infection. However, for patients with no such history, vasitis could go underdiagnosed because it mimics other diseases.

Infectious vasitis is associated with retrograde spread from cystitis, epididymitis, or prostatitis. Possible pathogens include *Escherichia coli* and *Haemophilus influenzae* [7]. Sexually-transmitted infections [4] and infections due to *Chlamydia trachomatis* [8] have also been reported. Rare pathogens such as *Mycobacterium tuberculosis* and Brucella have also been isolated [9]. Even though the urine culture is usually negative, we suggest routine urinalysis and culture before antibiotic treatment since concurrent urinary tract or surrounding organ infection may be present. The choice of initial empirical antibiotics can be based on sexual history, with consequent prescription of azithromycin or doxycycline for those with a history of recent sexual activities and quinolone for all others.

Sonography was the gold standard for diagnosis before the widespread use of high-resolution CT images. Yang et al. [10] reported retrospective sonographic findings in 12 patients with acute vasitis. Ten patients had lesions in the scrotum, one in the suprascrotal area, and one in both. Only one patient had undergone a vasectomy. A dilated vas deferens could be observed on a longitudinal greyscale sonography view. Heterogeneously hypoechoic lesions could be found within the scrotum under a transverse view. All patients showed increased blood flow on colour Doppler. Concomitant findings of epididymitis (11/12) and hydrocele (8/12) were also reported.

Some differential diagnoses should be considered before a definitive diagnosis of vasitis is made. First, an incarcerated inguinal hernia can present similar features in the acute setting. However, diameters of dilated bowel loops can be significant great in an inguinal hernia, with or without bowel gas (hyperechoic spots) [11]. A Valsalva manoeuvre performed during the ultrasound helps differentiate hernia from varicoceles. A multiple dilated pampiniform plexus of spermatic veins can be found as varicoceles, with a diameter greater than 3 mm, along with venous reflux during the Valsalva manoeuvre [12]. Varicoceles usually present on the left side, owing to the nutcracker effect on the left renal vein being compressed by the aorta and superior mesenteric artery. In addition, abscesses, benign and malignant tumours over the spermatic cord or the epididymis may be confused with vasitis under ultrasound [13, 14].

Although the diagnosis of vasitis entails differential diagnosis with a wide range of other clinical conditions, clinicians usually focus on ruling out only the acute ones, such as incarcerated inguinal hernia and testicular torsion. The initial diagnostic approach is with ultrasound. However, some studies revealed equivocal ultrasound results, thus advocating the use of further imaging modalities. Lin et al. [15] reported a case of vasitis with negative sonographic findings. Eddy et al. [16] and Kerkeni et al. [3] reported that three patients were initially misdiagnosed to have incarcerated inguinal hernias. Normal and symmetric testicular and epididymal sizes and blood flow were reported using sonography. Patel et al. [9] used magnetic resonance imaging (MRI) for diagnosis. Further imaging modalities are warranted and can be used if the ultrasound report is equivocal or does not correlate with the clinical findings. A contrast CT scan is valuable and can provide detailed information on the anatomy of each case; thus, an incarcerated inguinal hernia can easily be identified by this technique.

Unfortunately, few published papers have reported on the diagnosis of vasitis using CT scans since the previous approach to diagnosis was based only on ultrasound assessment. In a typical CT scan of vasitis [3, 4, 15–18], edematous changes over the spermatic cord and surrounding fat stranding are usually observed. Since contrast enhancement is dependent on the density of vessels, CT scans of inguinal hernias tend to enhance the bowel wall (peripheral predominance). In contrast, in vasitis, it tends to enhance the surrounding spermatic cord (central predominance) with peripheral inflammation. However, it is not exactly that the phase of contrast enhancement may be different from every intuition’s protocol, and the disease stage may affect the contrast distribution. As the thickened spermatic cord could mimic a bowel loop, the coronal view allows for establishing the correct diagnosis. Bowel loops have fluid or gas content, and the bowel wall is often dilated due to the proportional obstruction level. For vasitis, there is remarkable contrast enhancement on the spermatic cord, and thinner structures than bowel loops are displayed, with comparatively inferior and consistent diameters. In addition, there should be no traceable bowel loop herniation in the inguinal canal. Nevertheless, some other rare cases remain that may mimic the findings without bowel loops herniation on CT scan: 1. Amyand’s hernia due to an incarcerated appendix into the inguinal canal [19] 2. Inguinal herniation of mesenteric fat [20], hematoma [21] or cysts [22]. Compared with the equivocal findings that CT scans may yield in such instances, MRI has higher values on soft tissues so that the tract of the vas deferens can be clearly traced from the seminal vesicle. Vas deferens presents low intensity on both T1 and T2-weighted scans, while in vasitis, the edematous tissue can be adequately differentiated from the surrounding tissue. MRI also holds the advantage of no exposure to ionising radiation, especially in adolescents, who are the most prone to having vasitis. Even if characterised by many advantages, MRI is unfortunately still not readily applicable in the acute setting.

Compared with the typical clinical picture of vasitis reported in previous studies, our patient had enlargement and hyperattenuation over the spermatic cord and its adjacent vessels, with peripheral fat stranding on a CT scan. There was no intratubular content. The tubular structure could be traced back to the small bowel without interruption. Thus, the patient was misdiagnosed with omental or mesenteric incarceration in the inguinal area. That was the reason why we resolved to perform a diagnostic laparoscopy.

## Conclusions

To the best of our knowledge, this is the first case report of a patient with vasitis with a detailed medical record from physical examination to the definitive diagnosis through laparoscopy. Since vasitis is a rare disease, first and foremost, it is essential to differentiate it from acute severe conditions, such as testicular torsion and incarcerated inguinal hernia. Contrary to testicular torsion, there has been no formal report on decreased or absent blood flow on colour Doppler sonography in the case of incarcerated inguinal hernia. Both conditions share the same clinical features from the initial presentation to sonography. Our patient presented with omental adhesion in the inguinal region and swelling of the vas deferens, which mimicked omental incarceration on a CT scan due to contrast enhancement. Therefore, we performed diagnostic laparoscopy and only then could we formulate the correct diagnosis and treat the patient with appropriate antibiotics.

The present article purports to share the case of a patient with vasitis and atypical imaging findings on CT that did not help clarify the diagnosis.

## Data Availability

The data that support the findings of this study are available on request from the corresponding author. The data are not publicly available due to containing information that could compromise the privacy of research participants.

## Abbreviations

CT: Computed tomography
Beta-HCG: Beta-human chorionic gonadotropin
CRP: C-reactive protein
AFP: Alpha-fetoprotein
